# Potential Non-invasive Urine-Based Antigen (Protein) Detection Assay to Diagnose Active Visceral Leishmaniasis

**DOI:** 10.1371/journal.pntd.0002161

**Published:** 2013-05-30

**Authors:** Claudia Abeijon, Antonio Campos-Neto

**Affiliations:** 1 DetectoGen Inc., Grafton, Massachusetts, United States of America; 2 The Forsyth Institute, Cambridge, Massachusetts, United States of America; Institut Pasteur de Tunis, Tunisia

## Introduction

Visceral leishmaniasis (VL), also known as kala-azar, produces 500,000 new cases of disease per year around the world. VL is a serious and debilitating disease that affects economic productivity and quality of life, and is nearly 100% fatal if not treated promptly. VL occurs on four continents and is endemic in 47 countries, with approximately 200 million people at risk of infection. The number of cases is increasing, mostly due to deterioration of social and economic conditions and co-infection with HIV. Most of the approximately 50,000 deaths that occur each year due to VL happen in children (http://www.who.int/topics/leishmaniasis/en/).

There is no vaccine for VL, and the drugs that are most effective to treat the disease are toxic and painful, and prolonged treatment is required. For these reasons, prophylactic treatment, particularly for HIV co-infected patients, is not feasible.

The gold standard for diagnosis of VL is the aspiration of relevant tissues (biopsies) such as spleen, liver, bone marrow, or lymph nodes followed by direct visualization or isolation of the parasite in complex culture medium. Such sample collection is invasive and risky and requires trained personnel to identify the amastigote form of the parasite. Moreover, the sensitivity is variable and unsatisfactory, at about 60%–85% [1]–[4]. Conventional serological tests measure antibody responses to parasite antigens, but because antibodies can persist for years after cure, these tests cannot distinguish active VL from prior exposure [5]–[7]. In addition, these tests have low sensitivity in the rapidly growing populations that are co-infected with HIV [8], [9].

Therefore, for the above reasons, there is a critical unmet need for a rapid, sensitive, and specific test to identify people with active VL who need immediate treatment in order to save their lives and prevent the spread of VL to others in their communities.

## Development and Validation of a New Urine Test for Active VL

An ideal VL diagnostic should a) be able to differentiate active disease from formerly infected but healthy individuals, b) be amenable to monitoring treatment efficacy, and d) identify relapse or re-infection that is common in HIV co-infected individuals.

Recently, we introduced an innovative approach to directly identify antigens (proteins) from *Leishmania infantum* (etiological agent of VL) produced *in vivo* in patients with active VL [10]. By combining reverse-phase high-performance liquid chromatography (RP-HPLC) with mass spectroscopy, we identified in the patients' urine three distinct *L. infantum* proteins, namely *L. infantum* iron superoxide dismutase 1, NCBI accession number XP_001467866.1; *L. infantum* tryparedoxin 1, NCBI accession number XP_001466642.1; and *L. infantum* nuclear transport factor 2, NCBI accession number XP_001463738.1. The genes coding for these proteins were cloned, the recombinant proteins were produced and purified, and antibodies to them were produced in rabbits and chickens. The antibodies were purified from the sera and were used to develop capture enzyme linked immunosorbent assays (ELISA) designed to detect individually each of these *L. infantum* antigens in the urine of VL patients. In a sample of 20 irrefutably diagnosed VL patients, each *L. infantum* protein was identified in approximately 10–12 overlapping and non-overlapping urine samples. Moreover, in one sample no leishmanial antigen could be identified by any of the three assays. Moreover, none of the antigens were detected in the urine of patients with cutaneous leishmaniasis (CL), Chagas disease (CD), schistosomiasis (SC), or tuberculosis (TB). When compiled together, the urinary antigen detection ELISAs had a sensitivity of 89%, specificity of 100%, and limit of detection of 4–10 pg of antigen per ml of urine [10]. These samples were from patients from the area Teresina, PI, Brazil, and of Montes Claros city, MG, Brazil. Urine donation protocols were approved by the Investigational Review Board and the Ethics Committee of the Federal University of Piauí Medical School and Federal University of Minas Gerais, respectively.

**Figure 1:**
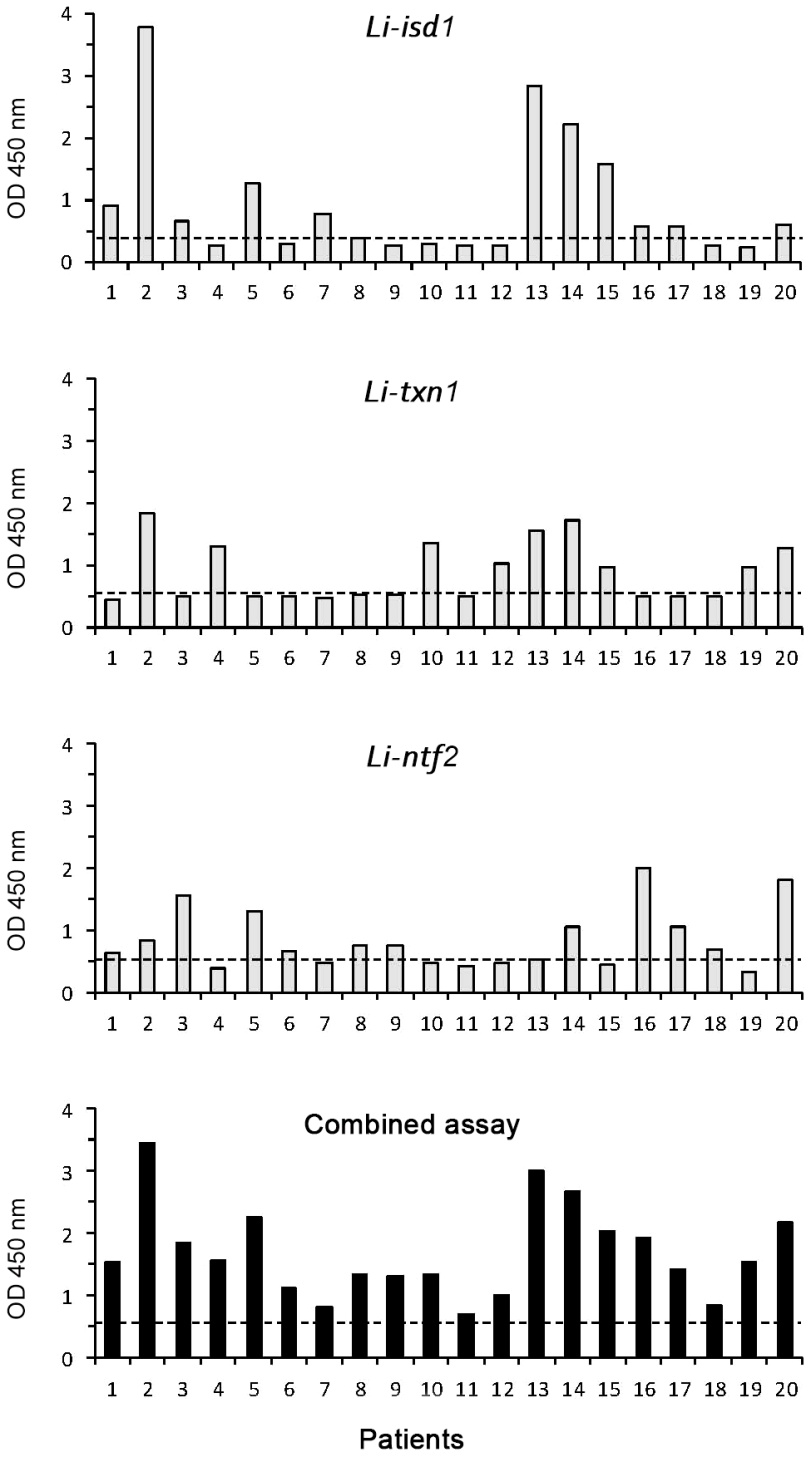
Antigen detection capture ELISA for the identification of the proteins *Li-isd1*, *Li-txn1*, and *Li-ntf2* in urine of VL patients and controls. Urine specimens were from VL patients and normal, healthy control subjects. The following predetermined capture antibodies concentrations were used to coat the ELISA plates: antigen affinity-purified IgY anti-*Li-isd1*, 100 ng/well; antigen affinity-purified rabbit anti-*Li-txn1* antibodies, 875 ng/well; and purified rabbit IgG anti-*Li-ntf2* antibody (2,000 ng/well), or the combination of the three antibodies/well. Samples from VL patients (*n* = 20) were from Teresina, PI, Brazil. Control samples were from healthy individuals from countries where VL is endemic who were living in the United States. Dashed lines represent the cutoff values calculated as described in the text. These are representative results of at least three experiments performed at different times with the same urine samples and same capture ELISA.

Here we report the results of a single assay assembled with the combination of the reagents used to individually detect the three *Leishmania* antigens (*Li-isd1*, *Li-txn1*, *and Li-ntf2*). This assay adds simplicity and sensitivity to the VL diagnostic test. As can be seen in Figure 1, confirming our previous results, 12/20 urine samples from VL patients were positive for the antigen *Li-isd1* with a cutoff (dotted line) of 0.432 calculated using the average of the ODs obtained from the urine specimens from 20 normal, healthy control subjects plus 3 SDs; 9/20 VL patient urine samples were positive for the antigen *Li-txn1* with a cutoff 0.610; and 11/20 VL patient urine samples were positive for *Li-ntf2* with a cutoff of 0.647. More importantly and surprisingly, 20/20 VL patient urine samples were positive for the combined capture ELISA assay. Interestingly, this result does not simply represent the detection of the individual antigens simultaneously in the combined assay. Thus, the urine of VL patient #11 was negative for each of the three individual assays but is clearly above cutoff for the combined assay. In addition, samples 6, 8, and 9, which were just borderline positive for antigen *Li-ntf2*, were clearly positive in the combined assay. Therefore, a synergistic effect was encountered by combining the three *Leishmania* antigen assays in a single capture ELISA. In summary, the combined assay was 100% sensitive in this pilot study using urine samples from patients diagnosed with VL using the gold standard test of bone marrow aspiration and parasite identification.

**Figure 2:**
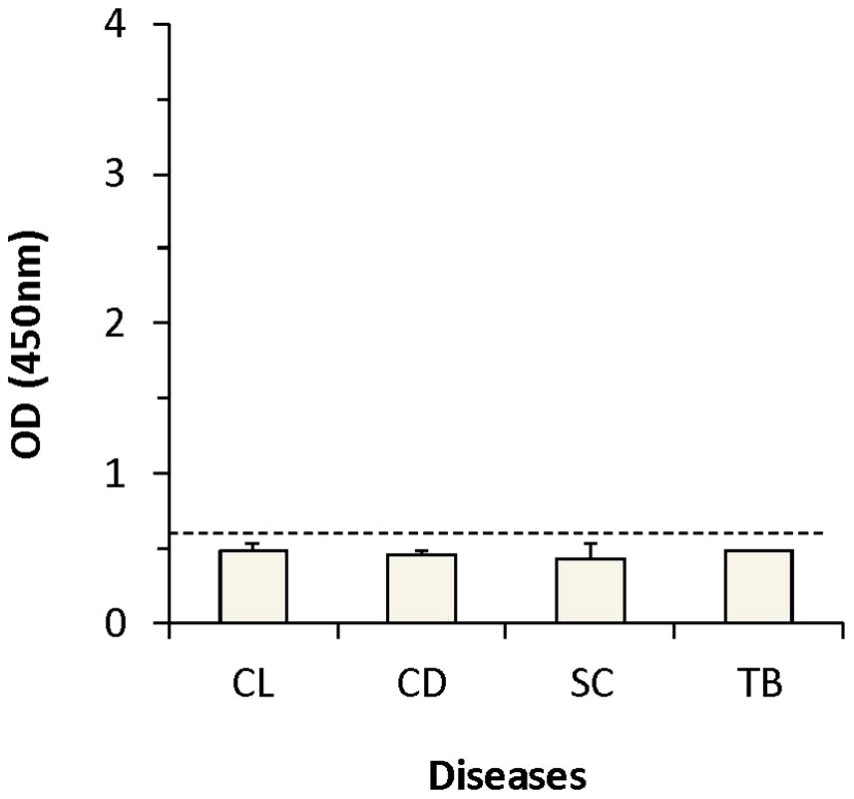
Specificity of the capture ELISA assembled with a combination of antibodies specific to *Li-isd1*, *Li-txn1*, and *Li-ntf2* antigens. Urine specimens were from patients with cutaneous leishmaniasis (CL) (*n* = 10), Chagas disease (CD) (*n* = 8), schistosomiasis (SC) (*n* = 14), or tuberculosis (TB) (*n* = 10). Assay performed using capture ELISAs assembled all three antibodies in a single well as described in the legend for Figure 1. Dashed line represents the cutoff values, which was calculated using the average of the ODs obtained from the urine specimens from 20 normal, healthy control subjects plus 3 SDs.

Next, we tested if the specificity of the combined capture ELISA remained at 100% as we described for the individual antigen assays [10]. Urine from patients with CL, CD, SC, and TB were tested with the combined capture ELISA and as expected were all negative (Figure 2), keeping the specificity of the assay at 100%. This is an important observation, as specificity of a clinically useful test for VL is critical because in areas endemic for VL, diseases like CL, CD, SC, and TB are prevalent as well.

Finally, it is relevant to mention that a latex agglutination test that detects leishmanial carbohydrate antigens in the urine of VL patients has been described for approximately 10 years. More recently, this test, known as KAtex, became commercially available in Europe (Kalon Biological Ltd, Guilford, UK). However, recent field studies with KAtex reported a wide variability in its sensitivity and specificity, 47.7%–100% and 84.3%–100%, respectively (Table 1). In contrast, the capture ELISA reported here overcomes these drawbacks (particularly the specificity problems) because it detects proteins instead of carbohydrates. As is well known, carbohydrates are very heterogeneous molecules, particularly when compared to the unique parasite proteins reported here. Moreover, the KAtex readout is based on degree of latex agglutination on a glass slide. This is of major concern due to the high subjectivity of this procedure.

**Table 1:** Comparison of the sensitivity/specificity of the new VL protein capture ELISA (this study) with that of available published data for a similar VL test that detects leishmanial carbohydrates (KATex).

Test	Sensitivity (%)	Specificity (%)	Reference
**VL protein capture ELISA**	100	100	This study
**VL carbohydrate detection test (KATex)**	75	100	Salam M et al. Trans R Soc Trop Med Hyg 105:269, 2011
	95.3	97	Ahsan M et al. Mymensingh Med J 19:335, 2010
	67	99	Sundar S et al. Trop Med Int Health 12:284, 2007
	57.4	84.3	Diro E et al. Trans R Soc Trop Med Hyg 101:908, 2007
	57	90	Chappuis F et al. Trop Med Int Health 11:31, 2006
	87	99	Hommel M et al. Am J Trop Med Hyg 73:269, 2005
	47.7	98.7	Rijal S et al. Trop Med Int Health 9:724, 2004
	68	100	Attar ZJ et al. Act Trop 78:11, 2001

## Overcoming Problems

Larger groups of VL caused by *L. infantum* (VL patients from the New World) and from VL caused by *Leishmania donovani* (VL patients from the Old World) will need to be tested. It is likely that our combined capture ELISA test will be equally useful for the diagnosis of VL patients from the Old World. This prediction is based on the fact that the three antigens discovered in the urine of patients with VL from the New World are 98% homologous to the same group of proteins produced by *L. donovani*.

In addition, in order to increase the sensitivity of the capture ELISA, we are in the process of preparing monoclonal antibodies against these antigens as well as large amounts of recombinant antigens for affinity purification of the polyclonal antibodies. These reagents should increase the sensitivity of the assay. Once this is achieved we will convert the current ELISA format to a point of care device utilizing the same pair of antibodies.

## Conclusion

We introduced a promising non-invasive antigen detection assay for the diagnosis of VL. This new assay is based on three *L. infantum* proteins previously identified in the urine of patients with VL. These antigens were used to generate antibodies that were used in combination to assemble a single capture ELISA for the clinical detection of these antigens in the urine of patients with VL. This new urine-based assay identified 20/20 VL patients and did not react with 62 urine samples obtained from control subjects. Although these encouraging results are based on a relatively small sample size, they warrant a future clinical trial to validate this important assay for the diagnosis of active VL. This trial will involve a large number of confirmed VL cases (including post-treated kala-azar), endemic controls, non-endemic controls, and other febrile diseases, particularly in areas where these infections are co-endemic with VL.

**Box 1. Advantages and disadvantages of the urine-based VL protein capture ELISA diagnostic test**

***Advantages***

- It is a non-invasive VL diagnostic test that can differentiate active disease from parasitic exposure and from cured VL patients.
- Should be a highly sensitive and specific test for VL.
- VL is fatal if not treated promptly; this test can aid a rapid diagnostic that will allow prompt therapy.
- Should be an important tool to follow-up therapy efficacy.
- Should be useful for the diagnosis of VL in VL/HIV-co-infected patients, as the conventional serological diagnosis of VL in these patients is problematic and not sensitive.

***Disadvantages***

- The current sensitivity might be slightly below the ideal for a clinical test that aims to diagnose patients with low parasite burden.
- Detection so far of only *L. infantum* (New World VL) antigens. Urine from VL patients caused by *L. donovani* (Old World VL) need to be tested.
- The current ELISA format is not suitable for use in rural scenarios with limited access to modern equipment. However, improvement of the antibody system (e.g., use of mAb) may circumvent this limitation.
